# ﻿Two new records and description of a new *Perinereis* (Annelida, Nereididae) species for the Saudi Arabian Red Sea region

**DOI:** 10.3897/zookeys.1196.115260

**Published:** 2024-04-01

**Authors:** Marcos A. L. Teixeira, Chloé Julie Loïs Fourreau, Juan Sempere-Valverde, Susana Carvalho

**Affiliations:** 1 Biological and Environmental Science and Engineering Division (BESE), King Abdullah University of Science and Technology (KAUST), Thuwal 23955-6900, Saudi Arabia; 2 Molecular Invertebrate Systematics and Ecology (MISE) Lab, Graduate School of Engineering and Science, University of the Ryukyus, Nishihara, Okinawa, Japan; 3 Laboratorio de Biología Marina/Estación de Biología Marina del Estrecho (Ceuta), Departamento de Zoología, Facultad de Biología, Universidad de Sevilla, Avda. Reina Mercedes s/n, 41012, Sevilla, Spain; 4 Marine Science Program, Biological and Environmental Science and Engineering Division, King Abdullah University of Science and Technology, Thuwal, 23955, Saudi Arabia

**Keywords:** Gulf of Aqaba, mtCOI-5P, NEOM, north-eastern Red Sea, Polychaeta, Saudi Arabia, taxonomy

## Abstract

Annelid biodiversity studies in the Red Sea are limited and integrative taxonomy is needed to accurately improve reference libraries in the region. As part of the bioblitz effort in Saudi Arabia to assess the invertebrate biodiversity in the northern Red Sea and Gulf of Aqaba, *Perinereis* specimens from intertidal marine and lagoon-like rocky environments were selected for an independent assessment, given the known taxonomic ambiguities in this genus. This study used an integrative approach, combining molecular with morphological and geographic data. Our results demonstrate that specimens found mainly in the Gulf of Aqaba are not only morphologically different from other five similar *Perinereis* Group I species reported in the region, but phylogenetic analysis using available COI sequences from GenBank revealed different molecular operational taxonomic units, suggesting an undescribed species, *P.kaustiana***sp. nov.** The new species is genetically close and shares a similar paragnath pattern to the Indo-Pacific distributed *P.helleri*, in particular in Area III and Areas VII–VIII. Therefore, we suggest it may belong to the same species complex. However, *P.kaustiana***sp. nov.** differs from the latter mainly in the shorter length of the postero-dorsal tentacular cirri, median parapodia with much longer dorsal Tentacular cirri, posteriormost parapodia with much wider and greatly expanded dorsal ligules. Additionally, two new records are reported for the Saudi Neom area belonging to *P.damietta* and *P.suezensis*, previously described only for the Egyptian coast (Suez Canal) and are distributed sympatrically with the new species, but apparently not sympatric with each other.

## ﻿Introduction

Based on genetic databases (i.e., BOLD and GenBank), and despite the recent advances in integrative studies focused on polychaetes (i.e., Nygren et al. 2010; Villalobos-Guerrero et al. 2021; Teixeira et al. 2023), there are still many taxonomic ambiguities and unidentified annelid species in some groups of Nereididae (i.e., Martin et al. 2021; Elgetany et al. 2022). *Perinereis* Kinberg, 1865 is one of the most diverse genera in this family, currently including between 97 (Wilson et al. 2023) to 106 (WoRMS Editorial Board 2024) valid species distributed worldwide. From these, approximately 16 species are reported for the Arabian Peninsula (Ocean Biodiversity Information System, OBIS; Mohammad 1971; Wehe and Fiege 2002). Due to apparent similar paragnath patterns, overall body features and lack of detailed systematic studies, *Perinereis* species are often problematic to identify to the species level (Bakken and Wilson 2005; Yousefi et al. 2011). This has led to informal denomination of species complexes and recognition of geographic morphs and varieties such as *P.cultrifera* (Grube, 1840) species group (type locality: Naples, Italy; Scaps et al. 2000) and the *P.nuntia* (Lamarck, 1818) species (type locality: Gulf of Suez, Egypt) group (Wilson and Glasby 1993; Glasby and Hsieh 2006; Sampértegui et al. 2013), both reported for the Red Sea (OBIS). Thanks to molecular data, it is now easier to screen for potential new species with apparent similar morphotypes. Recent evidence comparing populations from different regions has shown that when specimens differ genetically, further analysis of the diagnostic morphological features often leads to the recognition of distinct features that were previously overlooked (i.e., Sampértegui et al. 2013; Teixeira et al. 2022b). A recent review on meiofauna (Cerca et al. 2018) and recent polychaete studies (i.e., Abe et al. 2019; Tilic et al. 2019; Martin et al. 2020), including from Nereididae (Glasby et al. 2013; Sampieri et al. 2021; Teixeira et al. 2022a, b) also demonstrate that cryptic and pseudo-cryptic species often have geographically restricted distributions, with the range of cryptic species being smaller than the parent morphospecies.

The Egyptian side of the Red Sea has been the focus of an increasing amount of polychaete studies either reviewing existing species groups (i.e., Villalobos-Guerrero 2019) or describing new species that were previously considered cryptic (i.e., Elgetany et al. 2022). The northern Saudi Arabian Red Sea and Gulf of Aqaba, despite being expected to host a large biodiversity (Roberts et al. 2002; DiBattista et al. 2016), has seen comparatively few biodiversity studies involving molecular techniques, particularly for polychaetes. To address this gap, and document the invertebrate biodiversity of the region, a bioblitz was conducted in the Neom region (northern Saudi Arabian Red Sea and Gulf of Aqaba) to document the local biodiversity, with emphasis on mobile invertebrates and cryptobenthic fish. As part of this effort, this study used a molecular approach, combined with morphological and geographic data, to investigate *Perinereis* samples collected from marine intertidal and lagoon-like rocky environments of the northern Red Sea. In particular, we aimed to assess species distributions and to investigate whether specimens collected belonged to existing *P.cultrifera* group, *P.nuntia* group, to other similar *Perinereis* species reported for the region, or if new species were undescribed.

## ﻿Material and methods

### ﻿Sampling effort

The NEOM bioblitz sampling campaign surveyed 38 shallow and coral reef sites up to 25 meters depth and some intertidal habitats, along the northern region of the Saudi Arabian Red Sea and Gulf of Aqaba (Neom area). This initiative aims to initiate a biodiversity inventory of marine benthic invertebrates (mainly mobile) and cryptobenthic fish in the Red Sea using DNA barcoding and metabarcoding. Only intertidal marine and lagoon-like rocky environments were considered for the purpose of this study, in order to perform an independent assessment within *Perinereis*, given the known taxonomic ambiguities in several species within the genus from this particular habitat.

A total of 36 *Perinereis* specimens (atokous) were hand-collected on rocky shores, in coarse-grained sediments under cobbles and rocks. Specimens were found in Magna (centre of Gulf of Aqaba; 28°26'57.3"N, 34°45'35.4"E), Shushah Island (27°56'13.7"N, 34°54'36.1"E) and lagoon-like environments at Almojawah Bay (South of Gulf of Aqaba; 28°10'18.1"N, 34°38'57.6"E) and Duba (Al Muwaileh; 27°37'04.4"N, 35°31'26.7"E), in May 2023. Two specimens collected in the northern region of Portugal (Canto Marinho, 41°44'13.2"N, 8°52'33.6"W) belonging to *Perinereisoliveirae* (Horst, 1889), were previously collected by the first author of this work, and due to misidentifications with the *P.cultrifera* morphotype in genetic databases, were used in this study for comparison purposes.

Table 1 details the number of original specimens collected for each sampling location, which correspond to the same number of COI sequences analysed. The number of COI sequences from *Perinereis* species publicly available in GenBank, respective sampling area and references are also detailed in Table 1 and were used for comparison purposes. The collected Red Sea *Perinereis* specimens were deposited at NTNU University Museum, Trondheim, Norway (**NTNU-VM**, Bakken et al. 2024; vouchers: NTNU-VM-86010–NTNU-VM-86044). *Perinereisoliveirae* specimens are deposited at Biological Research Collection of the Department of Biology of the University of Aveiro (**CoBI** at **DBUA**; curated by Ascensão Ravara: aravara@ua.pt; vouchers: DBUA0002494.02.v01 and DBUA0002494.02.v02), Portugal. Specimens that were exhausted in the DNA analysis were assigned only with the Process ID from the BOLD systems (http://v4.boldsystems.org/), corresponding to MTPNO009-23 (Gulf of Aqaba, Magna). Some specimens were preserved in 96% ethanol and others in formalin with a respective sample tissue preserved in ethanol for molecular work (detailed in Suppl. material 1).

**Table 1.** Species, number of sequences (*n*), geographic location, and their respective GenBank COI accession numbers for the original material and sequence data used from other studies.

Species	GenBank COI	Region	Location	*n*	Reference
*Perinereiskaustiana* sp. nov.	PP279005, PP279009, PP279010, PP279017–PP279020, PP279029, PP279035	Red Sea	Saudi Arabia, Gulf of Aqaba (Magna)	9	This study
	PP279008, PP279025		Saudi Arabia, Shushah Island	2	
	PP279004		Saudi Arabia, Duba (Al Muwaileh)	1	
*Perinereissuezensis*	PP279006, PP279007, PP279015, PP279016, PP279021, PP279023, PP279024, PP279026–PP279028, PP279036, PP279038, PP279039		Saudi Arabia, Shushah Island	13	
	OP612968–OP612972		Egypt, Gulf of Suez	5	Elgetany et al. (2022)
*Perinereisdamietta*	PP279034		Saudi Arabia, Gulf of Aqaba (Magna)	1	This study
	PP279014, PP279037		Saudi Arabia, Duba (Al Muwaileh)	2	
	PP279002, PP279011–PP279013, PP279030–PP279033		Saudi Arabia, Gulf of Aqaba (Almojawah Bay)	8	
	OP610122–OP610126		Egypt, Gulf of Suez	5	Elgetany et al. (2022)
*Perinereisfayedensis*	OP605759–OP605763		Egypt, Gulf of Suez	5	
*Perinereisoliveirae*	PP279003, PP279022	NE Atlantic	Portugal, Canto Marinho	2	This study
“*Perinereiscultrifera*”	KR916909–KR916912		Portugal, Areosa Beach	5	Lobo et al. (2016)
*Perinereishelleri*	JX420256	Malaca Strait	Malaysia, Port Dickson	1	Glasby et al. (2013)
“*Perinereisnuntia*”	MH337359	Andaman Sea	India, Adaman and Nicobar Islands	1	Sivaraj and Thivikaran, unpublished
	JX420257	Java Sea	Indonesia, Pari Island	1	Glasby et al. (2013)
*Perinereismarionii*	OP347380	NE Atlantic	Great Britain, Plymouth	1	Teixeira et al. (2022b)
	OP347386		Portugal, Canto Marinho	1	
*Perinereisvallata*	HQ705192–HQ705196	South Pacific Ocean	Chile, Concepción	5	Sampértegui et al. (2013)
*Perinereiseuiini*	KY249122–KY249124	Yellow Sea	South Korea, Gusan-myeon	3	Park and Kim (2017)
	MN256544–MN256546	South China Sea	China, Xiamen	3	Xing and Zhang, unpublished
*Perinereisanderssoni*	MH143495, MH143498, MH143502, MH143503, MH143522	NW Atlantic	Brasil, Espirito Santo	5	Paiva et al. (2019)
*Alittavirens*	OP038747, OP038760, OP038799, OP038806, OP038851	North Sea	Sweden, Tjärnö-Salto canal	5	Teixeira et al. (2022a)

### ﻿DNA extraction, PCR amplification, and alignments

DNA sequences of the 5’ end of the mitochondrial cytochrome oxidase subunit I (mtCOI-5P) were obtained for all the collected *Perinereis* specimens and used for the main analysis. A representative number of specimens per location for the new species were also sequenced using the mitochondrial 16S rRNA and D2 region of nuclear 28S rRNA, for future reference purposes.

DNA extraction was performed using QuickExtract DNA Extraction Solution (Lucigen) with 50 µl of the reagent per Eppendorf. The tubes were then transferred to a heat block at 65 °C for 30 min and then an additional 2 min at 98 °C. Depending on the specimen size, only a small amount of tissue (i.e., a single parapodium) or the posterior end of the worm was used.

PCR reactions were performed using a premade PCR mix from VWR containing 10 µl per tube of Red Taq DNA polymerase Master Kit (2 mM, 1.1×), 0.5 µl of each primer (10 mM) and 1 µl of DNA template in a total 12 µl volume reaction. Table 2 displays the PCR conditions, primers and sequence lengths for the different markers. Amplification success was screened in a 1% agarose gel, using 1 μl of PCR product. Successful PCR products were then purified using the Exonuclease I and Shrimp Alkaline Phosphatase (ExoSAP-IT, Applied biosystems) protocol, according to manufacturer instructions. Cleaned up amplicons were sent to KAUST Sanger sequencing service for forward sequencing.

**Table 2.** Primers and PCR conditions used in this study.

Marker	Primer	Fragment	Direction (5’–3’)	PCR thermal cycling conditions	Reference
**COI**	PolyLCO	658bp	(F) GAYTATWTTCAACAAATCATAAAGATATTGG	1) 94 °C (1 min); 2) 5 cycles: 94 °C (40 s), 45 °C (40 s), 72 °C (1 min); 3) 35 cycles: 94 °C (40 s), 51 °C (40 s), 72 °C (1 min); 4) 72 °C (5 min).	Carr et al. (2011)
	PolyHCO		(R) TAMACTTCWGGGTGACCAAA RAATCA		
**16S**	16SAR-L	c.365bp	(F) CGCCTGTTTATCAAAAACAT	1) 94 °C (3 min); 2) 40 cycles: 94 °C (30 s), 52 °C (30 s), 72 °C (1 min); 3) 72 °C (7 min).	Kessing et al. (2004)
	16SANN-F		(F) GCGGTATCCTGACCGTRCWAAGGTA		
	16SBR-H		(R) CCGGTCTGAACTCAGATCACGT		
**28S**	28sC2	c.500bp	(F) ACTCTCTCTTCAAAGTTCTTTTC	1) 96 °C (4 min); 2) 45 cycles: 94 °C (30 s), 55 °C (30 s), 72 °C (1 min); 3) 72 °C (8 min).	Hassouna et al. (1984)
	28s-D2		(R) TCCGTGTTTCAAGACGG		

The obtained trace files were edited and aligned in MEGA software v. 11.0.10 (https://www.megasoftware.net/; Tamura et al. 2021). COI sequences were blasted in GenBank to access possible existing matches (NCBI; https://blast.ncbi.nlm.nih.gov/Blast.cgi). GenBank sequences batch for the original material are COI, PP279002–PP279039; 16S, PP264567–PP264572, PP264574, PP264575; 28SD2, PP264613, PP264614, PP264616. The dataset used for molecular analysis and its metadata can be accessed at the BOLD Systems under the project “*Perinereis* Saudi NEOM (DS-MTPNO)”, publicly available with the DOI: https://doi.org/10.5883/DS-MTPNO. The alignments (fasta and nexus format) for each individual marker and Suppl. material 1 are also publicly available online at Figshare: https://www.doi.org/10.6084/m9.figshare.25097756.

### ﻿Phylogenetic analysis and MOTU clustering

For comparison purposes, GenBank COI sequence data from *P.marionii* (Audouin & Milne Edwards, 1833); *P.vallata* (Grube, 1857); *P.helleri* (Grube, 1878); *P.cultrifera*; *P.euiini* Park & Kim, 2017; *P.anderssoni* Kinberg, 1865; *P.nuntia* (Lamarck, 1818); *P.fayedensis* Elgetany, Struck & Glasby, 2022; *P.suezensis* Elgetany, Struck & Glasby, 2022; *P.damietta* Elgetany, Struck & Glasby, 2022; and the outgroup *Alittavirens* (M. Sars, 1835) completed the final dataset (Table 1, Suppl. material 1). The phylogenetic analysis was performed through maximum likelihood (ML) for the entire dataset. Best-fit models were selected using the Akaike Information Criterion in MEGA. The phylogenetic relationship analysis was executed with 500 bootstrap runs using the General Time-Reversible model with gamma distributed rates and a portion of the sites invariable (GTR+G+I). The final version of the tree was edited with the software Inkscape v. 1.2 (https://www.inkscape.org).

Three delimitation methods were applied to obtain Molecular Operational Taxonomic Units (MOTUs): The Barcode Index Number (BIN), which makes use of the Refined Single Linkage (RESL) algorithm available only in BOLD (Ratnasingham and Hebert 2013); the Assemble Species by Automatic Partitioning (ASAP, Puillandre et al. 2021), implemented in a web interface (https://bioinfo.mnhn.fr/abi/public/asap/asapweb.html) with default settings using the Kimura-2-Parameter (K2P) distance matrix; lastly, the Poisson Tree Processes (bPTP; Zhang et al. 2013) performed in a dedicated web interface (https://species.h-its.org/), using the ML phylogeny obtained above, for 500000 MCMC generations and twenty-five percent of the samples discarded as burn-in.

The mean genetic distances for mtCOI (K2P; Kimura 1980) within and between MOTUs were calculated in MEGA.

### ﻿Morphological analysis

Specimens were studied using a Leica stereo microscope (model M205 C). Stereo microscope images were taken with a Flexacam C3 camera. Compound microscope images of parapodia and chaetae were obtained with a Leica DM2000 LED imaging light microscope, equipped with a Flexacam C3 camera, after mounting the parapodia on a slide preparation using Aqueous Permanent Mounting Medium (Supermount). Parapodial and chaetal terminology in the taxonomic section follows Bakken and Wilson (2005) with the modifications made by Villalobos-Guerrero and Bakken (2018). The final figure plates were edited with the software Inkscape v. 1.2.

For measuring length of dorsal ligules, not only the lengths of the tips were considered, but the proximal part of the ligules was also included (e.g., Conde-Vela and Salazar-Vallejo 2015; Villalobos-Guerrero and Carrera-Parra 2015; Teixeira et al. 2022b). Like Hutchings et al. (1991), a specimen is described as having a greatly expanded dorsal notopodial ligule posteriorly only if the dorsal ligule is more than two times as long as the ventral ligule. For analysis of variation, only complete specimens were considered; total length (**TL**) , length up to chaetiger 15 (**L15**) , width at chaetiger 15 (**W15**) were measured with a millimetre rule under the stereomicroscope. Number of chaetigers (**NC**) were also taken into consideration. TL was measured from anterior margin of prostomium to the end of the pygidium, and W15 were measured excluding parapodia. Measurements of the length of the antennae (**AL**), palps (**PL**), dorsal Tentacular cirri (**DCL**), dorsal ligule (**DLL**), ventral Tentacular cirri (**VCL**), ventral ligule (**VLL**), median ligule, the length and width of the head (**HL** and **HW**, respectively), and the length of all four tentacular cirri, including the longest one (postero-dorsal Tentacular cirri, **DPCL**), were also retrieved. Heterogomph falciger blade size comparison (short, long, and extra-long) based on Wilson et al (2023). Spiniger serration based on the comparison between *P.cultrifera* (lightly serrated) and *P.rullieri* (coarsely serrated) from Pilato (1974).

Paragnath counts were performed to compare patterns with other morphologically similar Group I *Perinereis* species (Hutchings et al. 1991). Pharynx paragnath terminology follows Bakken et al. (2009) and paragnath description of areas VII and VIII follow Conde-Vela (2018).

Terminology for molecular vouchers follows Pleijel et al. (2008) and Astrin et al. (2013). Overall description follows a similar structure to those of Villalobos-Guerrero (2019). Dates of sample collection follow the DD/MM/YY format.

## ﻿Results

### ﻿Phylogenetic analyses

**Figure 1.**
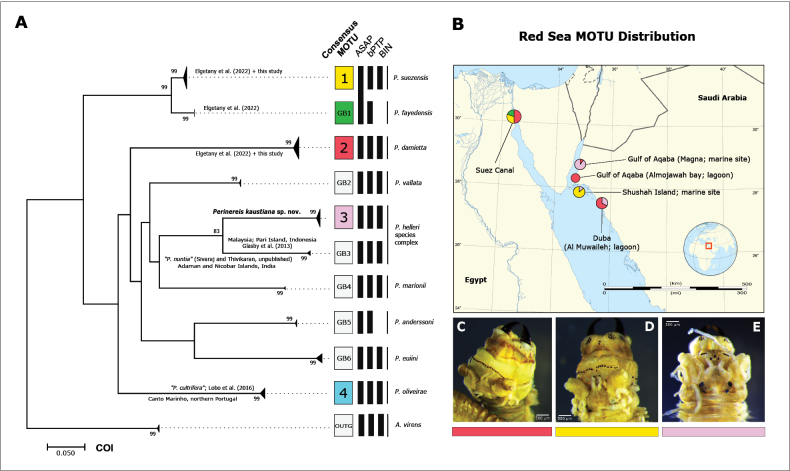
Phylogenetic tree and MOTU distribution for the three sampled Red Sea *Perinereis* species **A** maximum likelihood phylogeny based on COI sequences, with information regarding the different MOTU delineation methods. Numbered MOTUs (1–4) contain original sequences from *Perinereis* specimens analysed in this study; MOTUs “GB” are based on *Perinereis* sequences mined from GenBank; MOTU “OUTG” correspond to the rooted outgroup, *Alittavirens*. Bootstrap values lower than 80% not displayed **B** Red Sea MOTU distribution; each coloured pie corresponds to a unique species and respective abundance proportion; larger pie charts indicate higher number of sympatric species. Species from the Suez Canal based on mined GenBank sequences from Elgetany et al. (2022); abundance proportion based on type material **C***Perinereisdamietta*, focus on prostomium and pharynx, dorsal view, specimen NTNU-VM-86031 **D***Perinereissuezensis*, focus on prostomium and pharynx, dorsal view, specimen NTNU-VM-86032 **E***Perinereiskaustiana* sp. nov., focus on prostomium and pharynx, dorsal view, specimen NTNU-VM-86011. Scale bars: 500 μm (**C–E**).

The phylogenetic reconstruction recovered ten MOTUs of *Perinereis* (Fig. 1A), the delimitation of which are cohesively supported by the three species-delimitation tests applied, except for MOTU 1 and GB1, which are clustered together with the ASAP method. Sequences from *P.fayedensis* and *P.anderssoni* are not present in BOLD and have no associated BIN.

In this phylogenetic tree, *P.suezensis* (MOTU 1) and *P.fayedensis* (MOTU GB1) are sister to each other, and their lineage splits early compared to the other analysed species of *Perinereis*. The basal node support for the six molecular groups (2, 4, GB2, and GB4–GB6) is very low. The 36 Red Sea specimens sequenced in the present study clustered into three clades that correspond to *P.suezensis* (MOTU 1, Fig. 1D) and *P.damietta* (MOTU 2, Fig. 1C), as well as a molecular cluster exclusively harbouring sequences of specimens belonging to *P.kaustiana* sp. nov. (MOTU 3, Fig. 1E). The new species is sister to a group (MOTU GB3) including sequences attributed to *Perinereishelleri* from Malaysia (JX420256 from Glasby et al. (2013)), as well as two sequences probably misidentified as *Perinereisnuntia* from India and Indonesia (MH337359 from Sivaraj and Thivikaran (unpublished) and JX420257 from Glasby et al. 2013). This relationship was supported with 83% bootstrap replications. The genetic divergence between *P.kaustiana* sp. nov. and GB3 sequences group (19.9%, COI K2P) was higher than between the closely related but recently established as distinct species, *P.suezensis* and *P.fayedensis* (~ 6%, COI K2P). MOTU 4 grouped our sequences of *P.oliveirae* with *P.cultrifera* sequences from Lobo et al. (2016). Table 3 shows all the COI distances between and within the analysed taxa from Fig. 1.

**Table 3.** Mean intra (in bold) and inter-MOTU COI genetic distances (K2P; %), for the eleven analysed species/MOTUs in Fig. 1.

	1	2	3	4	5	6	7	8	9	10	11
***P.kaustiana* sp. nov.** (M. 3)	**1.0 ± 0.2**										
*P.helleri* (M. GB3)	19.9 ± 2.4	**0.5 ± 0.2**									
*P.suezensis* (M. 1)	25.8 ± 3.2	26.7 ± 3.2	**1.1 ± 0.2**								
*P.damietta* (M. 2)	23.8 ± 3.2	25.5 ± 3.2	25.9 ± 3.1	**0.8 ± 0.2**							
*P.fayedensis* (M. GB1)	25.3 ± 3.2	25.8 ± 3.2	5.8 ± 1.0	25.0 ± 3.0	**0.0 ± 0.0**						
*P.euiini* (M. GB6)	24.6 ± 3.2	25.5 ± 3.2	27.0 ± 3.4	25.3 ± 3.2	27.2 ± 3.4	**0.6 ± 0.2**					
*P.oliveirae* (M. 4)	25.8 ± 3.2	25.7 ± 3.2	25.4 ± 3.2	27.3 ± 3.4	24.8 ± 3.1	26.3 ± 3.3	**0.6 ± 0.2**				
*P.anderssoni* (M. GB5)	26.7 ± 3.4	24.6 ± 3.3	24.9 ± 3.1	26.4 ± 3.3	23.7 ± 3.0	22.3 ± 2.8	26.6 ± 3.4	**0.2 ± 0.1**			
*P.vallata* (M. GB2)	23.1 ± 3.0	22.0 ± 2.8	23.2 ± 3.0	22.0 ± 2.8	22.6 ± 2.9	24.1 ± 3.1	23.6 ± 2.9	24.3 ± 3.2	**0.1 ± 0.1**		
*P.marionii* (M. GB4)	25.9 ± 3.3	22.8 ± 3.0	25.2 ± 3.2	27.3 ± 3.4	23.8 ± 3.0	24.0 ± 3.2	23.6 ± 3.0	23.1 ± 3.0	21.1 ± 2.7	**0.3 ± 0.2**	
*A.virens* (OUTG)	29.3 ± 3.9	28.1 ± 3.5	27.5 ± 3.4	29.6 ± 3.6	27.5 ± 3.3	28.5 ± 3.5	27.6 ± 3.4	27.6 ± 3.5	26.3 ± 3.1	26.5 ± 3.2	**0.2** ± **0.1**

### ﻿Taxonomic account

**Family Nereididae Blainville, 1818**

**Genus *Perinereis* Kinberg, 1865**

#### ﻿ Perinereis kaustiana sp. nov.

Taxon classificationAnimaliaPhyllodocidaNereididae

D0B4FFBB-1B9A-53CE-BC6B-89F57F3105E4

https://zoobank.org/378AABC8-4C46-43FF-9A0F-5F6703B4A801

**Figure 2.**
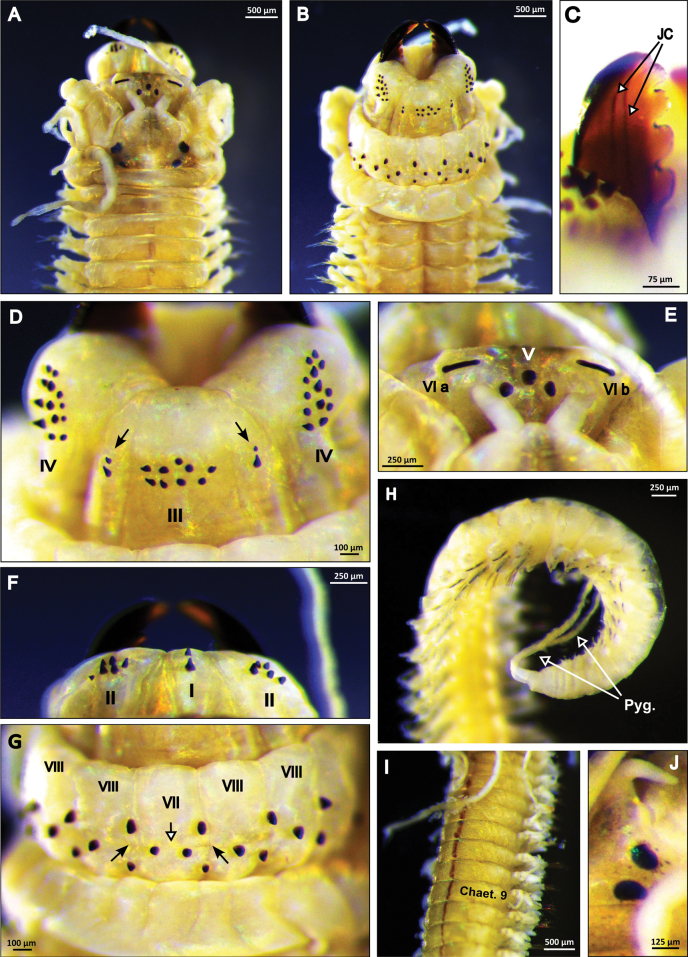
*Perinereiskaustiana* sp. nov. All pictures are from the holotype (NTNU-VM-86011) if not stated otherwise **A** anterior end, prostomium, dorsal view **B** anterior end, prostomium, ventral view **C** jaws and respective jaw canals (JC), dorsal view **D** pharynx, maxillary ring (Areas III and IV), ventral view; black arrows, lateral patches with two paragnaths each **E** pharynx, oral ring (Areas VI), dorsal view **F** pharynx, maxillary ring (Areas I and II), dorsal view **G** pharynx, oral ring (Areas VII–VIII), ventral view; black arrows, furrow regions; white arrows, ridge regions **H** posterior end; white arrows, pygidial Tentacular cirri, paratype (NTNU-VM-86015) **I** anterior body, tentacular cirri reaching chaetiger 9, paratype (NTNU-VM-86015) **J** worm’s eyes, right side, paratype (NTNU-VM-86015). Abbreviations: chaet., chaetiger; Pyg., Pygidium. Scale bars: 500 μm (**A, B, I**); 250 μm (**E, F, H**); 100 μm (**D, G**); 125 μm (**J**); 75 μm (**C**).

**Figure 3.**
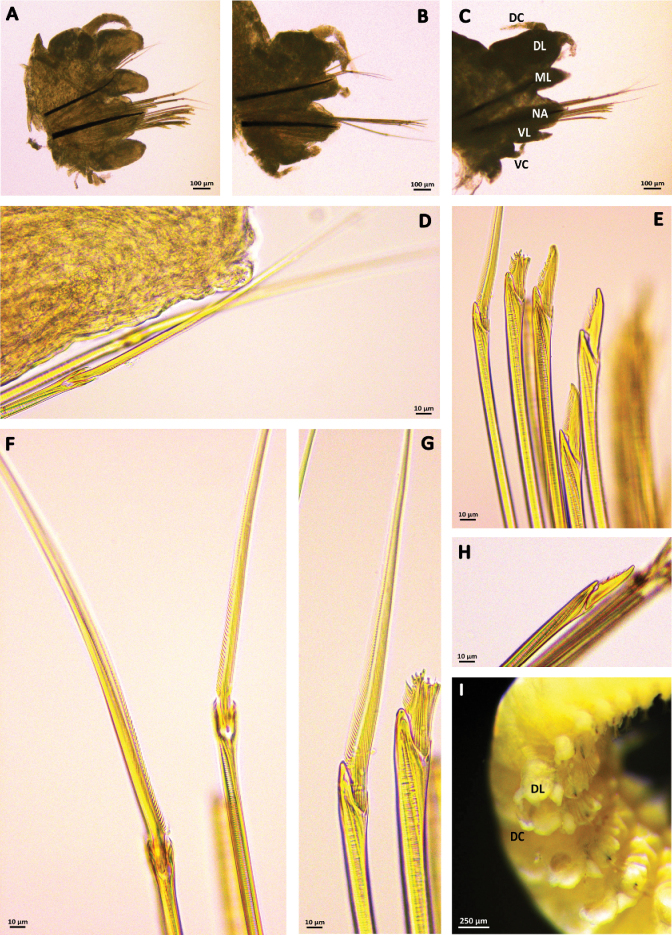
*Perinereiskaustiana* sp. nov. Parapodia and types of chaetae. All images are from the holotype (NTNU-VM-86011) **A** right parapodium, posterior view, chaetiger 9 **B** right parapodium, posterior view, chaetiger 46 **C** right parapodium, posterior view, chaetiger 96 **D** notochaetae: homogomph spiniger with lightly serrated blade, chaetiger 9 **E** neurochaetae, subacicular fascicle: heterogomph falcigers (centre) and heterogomph spinigers with lightly serrated blade (left), chaetiger 9 **F** neurochaetae, supra-acicular fascicle: homogomph spiniger with coarsely serrated blade, chaetiger 9 **G** neurochaetae, subacicular fascicle: heterogomph spiniger with lightly serrated blade, chaetiger 9 **H** neurochaetae, supra-acicular fascicle: heterogomph falciger, chaetiger 56 **I** posterior end, focused on chaetiger 97 and chaetiger 98. Abbreviations: DC, Dorsal Tentacular cirri; DL, Dorsal ligule; ML, Median ligule; NA, Neuroacicular ligule; VL, Ventral ligule; VC, Ventral Tentacular cirri. Scale bars: 250 μm (**I**); 100 μm (**A–C**); 10 μm (**D–H**).

Figs 1A, B, E ; 2A–J ; 3A–I

Nereis Perinereis helleri Grube, 1878: 81–82; Horst 1924: 172–173, pl 34, figs 3, 4.
Perinereis helleri Monro, 1931: 14–15, fig. 8a–c.; Russell 1962: 7; Rozbaczylo and Castilla 1973: 220–221; Hartmann Schröder 1979: 116.
Perinereis cultrifera var. helleri Fauvel 1932: 105–106.
Perinereis carniguina Grube, 1878: 87, pl 4, fig. 8.

##### Material examined.

***Holotype and hologenophore***: NTNU-VM-86011, Saudi Arabia (Red Sea), Gulf of Aqaba, Magna, 28°26'57.3"N, 34°45'35.4"E, intertidal, rocky beach among coarse-grained sand under rocks, collected by Marcos A. L. Teixeira and Chloé Julie Loïs Fourreau, 11/05/2023, GenBank (mtCOI): PP279020.

***Paratypes and paragenophores***: 7 specimens, NTNU-VM-86010, NTNU-VM-86012–NTNU-VM-86017, Saudi Arabia (Red Sea), Gulf of Aqaba, Magna, 28°26'57.3"N, 34°45'35.4"E, intertidal, rocky beach under rocks among coarse-grained sand, collected by Marcos A. L. Teixeira and Chloé Julie Loïs Fourreau, 11/05/2023, GenBank (mtCOI): PP279009–PP279010, PP279017–PP279019, PP279029, and PP279035.

##### Non-types.

2 specimens, NTNU-VM-86019, NTNU-VM-86020, Saudi Arabia (Red Sea), Shushah Island, 27°56'13.7"N, 34°54'36.1"E, intertidal, rocky beach under rocks among coarse-grained sand, collected by Marcos A. L. Teixeira, 05/05/2023. 1 specimen, NTNU-VM-86018, Saudi Arabia (Red Sea), Duba, Al Muwaileh, 27°37'04.4"N, 35°31'26.7"E, lagoon environment, intertidal, under rocks among coarse-grained sand, collected by Marcos A. L. Teixeira and Chloé Julie Loïs Fourreau, 18/05/2023. 1 specimen, MTPNO009-23, Saudi Arabia (Red Sea), Gulf of Aqaba, Magna, 28°26'57.3"N, 34°45'35.4"E, intertidal, rocky beach under rocks among coarse-grained sand, collected by Marcos A. L. Teixeira and Chloé Julie Loïs Fourreau, 11/05/2023.

##### Diagnosis.

Four pairs of tentacular cirri, postero-dorsal one reaching chaetiger 7–9; ratio of DPCL / HL = 3.6×. Eversible pharynx with one pair of dark brown curved jaws with seven or eight denticles; two longitudinal canals emerging from the pulp cavity, both in the mid-section of the jaw. Pharynx consisting of maxillary and oral rings with conical shaped paragnaths. Maxillary ring: Area I = 2 small paragnaths arranged in a longitudinal line. Area II = Cluster of 5–7 small paragnaths. Area III = central patch of nine small paragnaths, lateral patches with two small paragnaths each. Area IV = 13 small paragnaths arranged in wedge shape without any bars. Oral ring: Area V = a triangle of three large paragnaths. Area VI (a+b) = two narrow bar-shaped paragnaths, one on each side, displayed as a straight line. Areas VII–VIII = 20–24 small paragnaths in total; Area VII, ridge region with two transverse paragnaths, furrow regions with two longitudinal paragnaths each; Area VIII, ridge regions with one paragnath each, furrow regions with two longitudinal paragnaths each. Dorsal cirrus longer than ventral cirrus throughout the body; much longer in median chaetigers, ratio DCL / VCL = 2.8–3×. Ventral Tentacular cirri of median chaetigers shorter than ventral ligule, ratio of VCL / VLL = 0.7×. Dorsal ligule oval, ending tip gradually becomes thinner throughout the body; finger-like tip in median and posterior parapodia. Dorsal ligules of median chaetigers subequal to dorsal Tentacular cirri, tips shorter than dorsal Tentacular cirri. Posteriormost dorsal ligules greatly expanded (3× the length of the ventral ligule) and visibly much wider (2.5–3× the width of median ligule) than anterior and median ones (2× the width of median ligule). Pygidial Tentacular cirri as long as last 12–14 chaetigers.

##### Molecular Data.

MtCOI-5P, 16S, and 28S sequences as in specimens NTNU-VM-86010–NTNU-VM-86020 and MTPNO009-23 (Table 1; Suppl. material 1). GenBank accession numbers: PP279004, PP279005, PP279008–PP279010, PP279017–PP279020, PP279025, PP279029, PP279035 (mtCOI- 5P); PP264567–PP264572, PP264574, PP264575 (16S); PP264613, PP264614, PP264616 (28S-D2). *Perinereiskaustiana* sp. nov. clearly differs from the remaining species of the COI phylogeny, grouping in MOTU 3 (Fig. 1A). No GenBank BLAST match to date. Sister species with *P.helleri*. Intraspecific mtCOI-5P mean distances below 1%. Interspecific mtCOI-5P mean distances to the closest and distant neighbour are 19.9% (K2P, *P.helleri*) and 26.7% (K2P, *P.anderssoni*) respectively. DOI for the species’ Barcode Index Number (BIN): https://doi.org/10.5883/BOLD:AFJ4260.

##### Distribution and habitat.

Confined to the northeastern Red Sea (Duba, Shushah Island) and Gulf of Aqaba (Magna) so far. ***Type locality***: Saudi Arabia, Gulf of Aqaba: Magna region (marine site), 28°26'57.3"N, 34°45'35.4"E. Specimens collected both in lagoon-like environments and fully marine sites in rocky areas, usually among coarse-grained sand under rocks. Apparently more abundant and easier to find in marine sites from the Gulf of Aqaba. Can be found in sympatry with *P.damietta* (Fig. 1B, C) and *P.suezensis* (Fig. 1B, D). The latter two species as described by Elgetany et al. (2022).

##### Etymology.

The species designation pays tribute to the King Abdullah University of Science and Technology (KAUST) in Saudi Arabia, a globally recognized graduate-level research institution. This naming honours KAUST’s substantial and enduring contributions to marine science, particularly in advancing our understanding of the Red Sea over the course of more than a decade. Through its dedicated research efforts, KAUST has significantly enriched the scientific community’s knowledge of this unique marine environment.

##### Description.

Specimens used: NTNU-VM-86011 (holotype) and NTNU-VM-86015 (paratype), both preserved in ethanol 96%, stored at NTNU University Museum (Norway, NTNU-VM).

***Body/measurements***: Body with a prominent dorsal blood vessel (Fig. 2I); stout anteriorly, posteriorly gradually tapering toward pygidium. Colour in preserved specimens is yellowish-brown. Holotype, NTNU-VM-86011, large specimen, complete, TL = 55 mm, L15 = 7 mm, W15 = 2.12 mm, with 115 chaetigers. Paratype, NTNU-VM-86015, small specimen, complete, TL = 24 mm, L15 = 5 mm, W15 = 1.06 mm, with 85 chaetigers.

***Head*** (Fig. 2A, B, E, J): Prostomium pyriform, 1.2× wider than long; 2.5× longer than antennae. Palps with a round or conical palpostyle (Fig. 2A); palpophore longer than wide, subequal to the entire length of prostomium. Antennae separated, gap half of antennal diameter (Fig. 2E); tapered, less than half the length of the palpophore. Eyes black, anterior and posterior pairs well separated (Fig. 2J). Anterior pair of eyes oval shaped, as wide as antennal diameter; posterior pair of eyes round or oval shaped, subequal width to anterior pair. Distance between the anterior eyes 1.25× longer than posterior ones. Nuchal organs covered by the tentacular belt.

***Tentacular cirri***: Tentacular cirri longer than mid body width. Tentacular cirri pattern: postero-dorsal Tentacular cirri twice longer than antero-dorsal ones; postero-dorsal reaching chaetiger 7–9 (Fig. 2I). Antero-dorsal Tentacular cirri reaching chaetigers 3 and 4; 1.7× longer than palpophore. Antero-ventral Tentacular cirri 1.4× shorter than postero-ventral ones; antero-ventral shorter than palpophore. Dorsal cirrophores wrinkled, cylindrical.

***Pharynx***: Pair of dark brown curved jaws with 7–8 denticles; two longitudinal canals emerging from the pulp cavity, both in the mid-section of the jaw (Fig. 2C). Pharynx consisting of maxillary and oral rings with conical shaped paragnaths (Fig. 2A, B). Maxillary ring: Area I = two small paragnaths arranged in a longitudinal line (Fig. 2F). Area II = Cluster of 5–7 small paragnaths (Fig. 2F). Area III = central patch of nine small paragnaths, lateral patches with two small paragnaths each (Fig. 2D). Area IV = 13 small paragnaths arranged in wedge shape without any bars (Fig. 2D). Oral ring: Area V = a triangle of three large paragnaths (Fig. 2E). Area VI (a+b) = two narrow bar-shaped paragnaths, one on each side, displayed as a straight line (Fig. 2E). Areas VII–VIII = 20–24 small paragnaths in total; Area VII, ridge region with two transverse paragnaths, furrow regions with two longitudinal paragnaths each (Fig. 2G); Area VIII, ridge regions with one paragnath each, furrow regions with two longitudinal paragnaths each (Fig. 2G).

***Notopodia***: Dorsal Tentacular cirri slender, tapering, subequal to dorsal ligule in anterior (Fig. 3A) and median (Fig. 3B) parapodia, 1.8× shorter in posterior ones (Fig. 3C); Tentacular cirri longer than proximal part of dorsal ligule in anterior and median parapodia, 1.4× shorter in posterior ones. Dorsal Tentacular cirri longer than ventral one throughout the body, much longer in median chaetigers; 1.7× longer in anterior and posterior parapodia, 2.4× in median ones. Dorsal ligules oval, ending tip gradually becomes thinner throughout the body; finger-like tip in median and posterior parapodia (Fig. 3A–C). Dorsal ligules 1.4× longer and twice as wider as median ligules in anterior parapodia (Fig. 3A), 1.6× longer and twice wider in median ones (Fig. 3B), twice longer and 2.5–3.0× wider than median ligules in posterior parapodia (Fig. 3C). Posteriormost dorsal ligules greatly expanded; 3× the length of the ventral ligule; visibly much wider (2.5–3×) than median ligule (Fig. 3C, I). Distal part of dorsal ligules slightly longer than proximal one in anterior and median parapodia, 1.5× shorter in posterior ones.

***Neuropodia***: Ventral Tentacular cirri slender with tapering tip, 1.35× shorter throughout the body (Fig. 3A–C). Neuroacicular ligules subequal to ventral ligule in anterior parapodia, 1.3–1.4× longer in median and posterior ones. Ventral ligules oval in anterior parapodia, gradually becomes thinner throughout the body with a tapering tip; ventral ligules 1.4× shorter than dorsal ligules in anterior parapodia (Fig. 3A), twice shorter in median ones (Fig. 3B), 2.5–3× shorter in posterior parapodia (Fig. 3C).

***Chaetae***: Notochaetae with homogomph spinigers; spinigers with lightly serrated blade, evenly spaced (Fig. 3D), numerous and present throughout the whole body. Neurochaetal supra-acicular fascicle with homogomph spinigers (Fig. 3F) and heterogomph falcigers (Fig. 3H) present throughout the whole body; spinigers with coarsely serrated blade, present in the dorsal most position; falcigers with slender serrated long blade (Fig. 3H). Neurochaetal subacicular fascicle with heterogomph spinigers (Fig. 3G) and heterogomph falcigers (Fig. 3E) both present throughout the whole body; spinigers with lightly serrated blades (Fig. 3G); falcigers similar to supra-acicular ones (Fig. 3H), present in the ventral most position.

***Pygidium***: With a pair of long cylindrical slender anal Tentacular cirri, as long as last 12–14 chaetigers (Fig. 2H).

##### Remarks.

Some nereidid species groups can have similar morphological features, including paragnath patterns, that may cause misidentifications. The new species COI clade revealed no GenBank match based on the BLAST tool. *Perinereiskaustiana* sp. nov. and a sequence belonging to a specimen from Malaysia identified as *P.helleri* (type locality: Bohol, Philippines) not only are sister to each other and phylogenetically close (Fig. 1A; 19.9 ± 2.4% K2P COI distance), but they also seem to share the same paragnath sizes, shapes and patterns (Park and Kim 2017: 255, fig. 4e; sampled in South Korea; no molecular data available), including in Area III, with the presence of lateral patches with two paragnaths each (Fig. 2D) and the same paragnath arrangements in the furrow and ridge regions of Areas VII–VIII (Fig. 2G). This makes them morphologically very similar and possibly belonging to the same cryptic complex, which could range from the Red Sea to the Indo-Pacific based on the available COI data. However, *P.kaustiana* sp. nov. seems to differ from *P.helleri* in some key features: shorter postero-dorsal tentacular cirri, reaching up to chaetiger 9, instead of the reported chaetiger 16 for *P.helleri*; median parapodia with much longer dorsal Tentacular cirri (3×) compared to ventral one; posteriormost parapodia with much wider dorsal ligule (2.5–3.0×) than the median ligule (Fig. 3C, I) and dorsal ligule greatly expanded (3× longer than ventral ligule). Based on parapodia drawings from Hutchings et al. (1991: 255, fig. 9; Syntype ZMB Q3464), the ratio between dorsal and ventral Tentacular cirri in *P.helleri* is subequal to slightly longer than ventral Tentacular cirri throughout the body and posteriormost dorsal ligules with double the width of median ones and slightly expanded (up to 2× the length of the ventral ligules; Table 4). Furthermore, *P.helleri* from Hutchings et al. (1991) does not seem to possess ligules with finger-like ending tips.

**Table 4.** Comparison between selected characters in the most morphologically similar species to *P.kaustiana* sp. nov., reported for the Arabian Peninsula and Mediterranean Sea and lacking DNA data. The Indo-Pacific *P.helleri* is also included. Morphological details of paragnath patterns for *P.cultrifera* and *P.rullieri* species complexes also includes partial data from topotypical specimens belonging to the private collection of the first author, to be published in the forthcoming future.

Characters	*P.kaustiana* sp. nov.	*P.helleri* (Grube, 1878)	*P.cultrifera* (Grube, 1840)	*P.rullieri* Pilato, 1974
Colouration	Yellowish to yellowish brown	Creamy pink or pale brown	Yellowish brown to dark brown; faint narrow transverse pigmented bands on several anterior chaetigers	Yellowish brown to dark brown
Paragnaths Area I	2 small paragnaths arranged in a longitudinal line	Usually 2 small paragnaths arranged in a longitudinal line; occasionally 1	Usually 2 paragnaths arranged in a longitudinal line; occasionally 1	Usually one; may have 2 paragnaths arranged in a longitudinal line
Paragnaths Area II	Cluster of 5–7 small paragnaths	Cluster of 4–17 small paragnaths	Diagonal band of 3–15 large paragnaths	Cluster of 3–15 small paragnaths
Paragnaths Area III	Central patch of 9 small paragnaths + lateral patches with 2 paragnaths each	Central patch of 11–20 small paragnaths + lateral patches with 2 or 3 paragnaths each	Central patch of 5–11 large paragnaths, lateral patch absent	Central patch of 5–16 small paragnaths + lateral patches (may be present only on a single side) with 1 paragnath each
Paragnaths Area IV	13 small paragnaths arranged in wedge shape without any bars.	10–19 small paragnaths arranged in wedge shape, without any bars.	6–20 paragnaths arranged in wedge shape, without any bars.	10–25 paragnaths arranged in wedge shape without any bars.
Paragnaths Area V	Triangle of 3 paragnaths	Triangle of 3 paragnaths	Variable. Usually a triangle of 3 paragnaths; may occasionally display a single paragnath or have 4 paragnaths arranged in rhomboid shape	Usually a triangle of 3 paragnaths; may occasionally display a single paragnath
Paragnaths Areas VI (a+b)	2 narrow, straight bars	2 narrow, straight bars	Variable, usually 2 broader and straight bars; may have narrow and/or arcuate and/or short bars	Variable, usually 2 narrower and straight bars; may be very short
Paragnaths Areas VII, VIII	Area VII, ridge region with 2 transverse paragnaths, furrow regions with 2 longitudinal paragnaths each; 20–24 total.	Area VII, ridge region with 2 transverse paragnaths, furrow regions with 2 longitudinal paragnaths each; 21–40 total	Usually arranged in two regular rows of large paragnaths; 20–50 total	Usually arranged in two irregular rows of small paragnaths; 20–40 total
Postero-dorsal Tentacular cirri	Medium sized, reaching up to chaetiger 9	Long sized, reaching up to chaetiger 16	Small sized, reaching up to chaetigers 4 and 5	Medium sized, reaching up to chaetigers 6–8
Homogomph spiniger serration (neurochaetal supra-acicular fascicle)	Coarsely serrated	No data	Lightly serrated	Coarsely serrated
Heterogomph falcigers	Present with long blades	Present with long blades	Present with short blades	Present with long blades
Parapodia	Posteriormost dorsal ligules greatly expanded (3× longer than ventral ligule) and much wider (2.5–3× wider than median ligule) than in anterior and median ones. Median dorsal Tentacular cirri much longer than ventral ones (ratio 2.8–3×)	Posteriormost dorsal ligule expanded (1.9–2× longer than ventral ligule). Dorsal Tentacular cirri subequal to ventral Tentacular cirri throughout the body	Posteriormost dorsal ligule expanded (1.5–1.8× longer than ventral ligule); may be greatly expanded (> 2×)	Posteriormost dorsal ligule expanded (1.5–1.8× longer than ventral ligule); may be greatly expanded (> 2×)
Type locality	Gulf of Aqaba, Saudi Arabia (Red Sea)	Philippines, Bohol (Pacific)	Naples, western Italy (Mediterranean)	Sicily, Eastern Italy (Mediterranean)
Reference	This study	Mohammad 1971; Hutchings et al. 1991	Hutchings et al. 1991; Pilato 1974	Pilato 1974

Other species with similar paragnath patterns are *Perinereisanderssoni* (Kinberg 1865: 167–179; Park and Kim 2017: 255, fig. 4d) and *Perinereisrullieri* (Pilato 1974: 25–36, figs 1–4), which share the same small sized paragnaths as *P.kaustiana* sp. nov., but instead the former two species possess only one paragnath in each lateral patch of Area III and paragnaths in Areas VII and VIII are usually arranged in two regular rows, without any discernible pattern in the furrow or ridge regions. *Perinereisanderssoni* is reported in the Atlantic region of the American continent (type locality: Rio de Janeiro, Brazil), while *P.rullieri* is apparently restricted to the Mediterranean Sea (type locality: between Aci Trezza and Augusta, eastern coast of Sicily, Italy). Moreover, the morphological similar lineages found within the *Perinereiscultrifera* (Grube 1840: 74, fig. 6; Hutchings et al. 1991: 253–254, fig. 8a–c) species complex, including *P.euiini* (Park and Kim 2017: 252–260, figs 1, 2, 4a, b, 5, tables 1, 4, described for South Korea), are different from *P.kaustiana* sp. nov. due to the overall larger paragnath sizes, lack of any lateral patches in Area III, and the presence of shorter heterogomph falcigers (Park and Kim 2017: 254, fig. 2L). Specimens of *Perinereiscultrifera* from Lobo et al. (2016) were misidentified and are in fact *P.oliveirae* (Horst 1889: 38–45, plate 3; Fauvel 1923: 354, fig. 138 e–k), the latter characterised by the presence of three paragnaths in lateral patches in Area III, while this feature is absent in *P.cultrifera*. *Perinereisoliveirae* is described for the northern Iberian Peninsula, having also very long bar-shaped paragnaths in Areas VI and very short tentacular cirri compared to length of the head (reaching chaetigers 1 and 2). These features were confirmed based on the two *P.oliveirae* specimens from this study and samples from the private collection of the first author of this study.

Apart from the above-mentioned species, based on WoRMS (https://www.marinespecies.org/; Read and Fauchald 2024), OBIS (https://mapper.obis.org/), the *Perinereis* checklist from Mohammad (1971) for the Arabian Gulf and the annotated checklist of polychaete species around the Arabian Peninsula from Wehe and Fiege (2002), there are five additional *Perinereis* species with just a single bar-shaped paragnath on each side of Area VI (*Perinereis* species Group I, Hutchings et al. 1991) reported for the Arabian Peninsula: *P.perspicillata* (Grube, 1878) (Bonyadi-Naeini et al. 2018: 1973, table 4); *P.iranica* Bonyadi-Naeini, N. Rastegar-Pouyani, E. Rastegar-Pouyani, Glasby & Rahimian, 2018: 1965–1976, figs 2, 3, table 4; *P.obfuscata* (Grube, 1878) (Bonyadi-Naeini et al. 2018: 1973, table 4); *P.striolata* (Grube, 1878) (Bonyadi-Naeini et al. 2018: 1973, table 4) and *P.floridana* (Ehlers, 1868: 269–748, pls XII–XXIV), as interpreted by Bonyadi-Naeini et al. (2018: 1972–1973, table 4). None of the above species share the same morphotype as *P.kaustiana* sp. nov., differing in the paragnath numbers and arrangement, as well as the length of the postero-dorsal Tentacular cirri and types of chaetae, as described in the taxonomic key below. Additionally, four *Perinereis* Group I species reported mainly for the Mediterranean Sea were added to the key due to geographical proximity for comparison purposes: *Perinereiscultrifera*, *Perinereisrullieri*, *Perinereismacropus* (Claparède, 1870: 444–448, pl. VIII, fig. 1), and *Perinereistenuisetis* (Fauvel, 1915) (Guerne and Richard 1916: 88–92, pl. VII, figs 1–10; Mahcene et al. 2023). A comparison between selected characters in the most morphologically similar species to *P.kaustiana* sp. nov., some lacking DNA data and reported for the Arabian Peninsula and Mediterranean Sea, are summarised in Tables 4, 5 (including *P.helleri*).

**Table 5.** Comparison between selected characters in the most morphologically similar species to *P.kaustiana* sp. nov., reported for the Arabian Peninsula and Mediterranean Sea and lacking DNA data.

Characters	*P.iranica* Bonyadi-Naeini et al. (2018)	*P.perspicillata* Grube, 1878	*P.macropus* (Claparède, 1870)	*P.floridana* (Ehlers, 1868)
Colouration	Creamy with orange pigmentation in anterior body	No data	Greenish; white pigmented dots in prostomium and anterior region (based on the drawings)	No data
Paragnaths Area I	Cluster of 4–6 paragnaths	Cluster of 6–8 paragnaths	Cluster of 4 paragnaths	1 large paragnath.
Paragnaths Area II	12–15 paragnaths	Cluster of paragnaths	20–25 paragnaths arranged in wedge shape	Group of paragnaths in three oblique rows
Paragnaths Area III	Central patch of 30–45 paragnaths, lateral patch absent	Central patch, lateral patch absent	Central patch of 30–35 paragnaths + lateral patches with 4 paragnaths each	Group of very small paragnaths in three parallel rows
Paragnaths Area IV	40–47 paragnaths arranged in wedge shape without any bars.	Cluster of very dark paragnaths	25–27 paragnaths arranged in an inverse triangle	Group of paragnaths in four parallel rows, the last shorter than the others and ending in a cluster in the central corner
Paragnaths Area V	5 paragnaths in one row, median one larger than others	Triangle of 3 paragnaths	5 paragnaths in one row + single middle paragnath on top of the row	1 large paragnath
Paragnaths Area VI (a+b)	2 long, arcuate bars	2 large transversal bars	2 slightly arcuate transversal bars	1 single, broad, flat, somewhat triangular paragnath
Paragnaths Areas VII and VIII	3 rows, two distal most rows composed of large paragnaths and proximal row comprising small paragnaths; 25–31 total	Central group with three rows, lateral ones with one row	Band of 50–55 paragnaths	Group of large paragnaths in two distinct rows
Postero-dorsal Tentacular cirri	Very small, reaching up to chaetiger 2	No data	Very small, reaching up to chaetiger 2	Extend back to chaetiger 5
Homogomph spiniger serration (neurochaetal supra-acicular fascicle)	No data	No data	No data	No data
Heterogomph falcigers	Present with short blades	No data	Short blades	Present with short, sickle-shaped blades
Parapodia	Posteriormost dorsal ligule greatly expanded	No data	Posteriormost dorsal ligule not expanded	No data
Type locality	Iran (Persian Gulf)	Philippines (Pacific)	Naples, western Italy (Mediterranean)	Caribbean Sea
Reference	Bonyadi-Naeini et al. 2018	Fauvel 1911; Mohammad 1971	Claparède 1870	Wesenberg-Lund 1949; Bonyadi-Naeini et al. 2018

### ﻿Key to the *Perinereis* species Group I reported for the Arabian Peninsula and Mediterranean Sea, including the Indo-Pacific ingroup *P.helleri*

**Table:** 

1	Area V, usually a triangle of 3 paragnaths or more	**2**
–	Area V, a single paragnath or absence	**8**
2	Area I, a small cluster of 4–8 paragnaths	**3**
—	Area I, 1 to 3 paragnaths; if more than 1, arranged in a longitudinal line	**4**
3	Area III, cluster of paragnaths, lateral patches absent; Area VI, a large transversal bar	***P.perspicillata***
—	Area III, cluster of paragnaths, lateral patches present, 4 paragnaths each; Area VI, a short arcuate bar	***P.macropus***
4	Large paragnath sizes; Area III, lateral patches absent. Short heterogomph falcigers	**5**
—	Small paragnath sizes; Area III, lateral patches with 1 or 2 paragnaths each. Long heterogomph falcigers	**6**
5	Area V, one row of 5 paragnaths, median one larger than others; Area VI, a long clear arcuate bar	***P.iranica***
—	Area V, a triangle of 3 paragnaths; Area VI, a short straight bar, may be slightly arcuate	***P.cultrifera* (complex)**
6	Area III, lateral patches with 1 paragnath each (may be present only on a single side)	***P.rullieri* (complex)**
—	Area III, lateral patches with 2 paragnaths each	**7**
7	Length of postero-dorsal Tentacular cirri extends back to chaetiger 16 (range 8–16). Dorsal Tentacular cirri subequal to slightly longer than ventral one throughout the body. Posteriormost dorsal ligules expanded. Indo-Pacific variant	***P.helleri***
—	Length of postero-dorsal Tentacular cirri extends back to chaetigers 7–9. Dorsal Tentacular cirri of median segments much longer than ventral Tentacular cirri (DCL / VCL = 2.8–3×). Posteriormost dorsal ligules greatly expanded. Red Sea variant	***P.kaustiana* sp. nov.**
8	Area V, absence of paragnaths. Homogomph falcigers present; heterogomph falcigers absent	***P.tenuisetis***
—	Area V, a single paragnath. Homogomph falcigers absent; heterogomph falcigers usually present^1^	**9**
9	Area I, 1 large paragnath	***P.floridana***
—	Area I, a small cluster of 3–9 paragnaths	**10**
10	Tentacular cirri reaching backwards to chaetiger 1	***P.obfuscata***
—	Tentacular cirri reaching backwards to chaetigers 6 and 7	***P.striolata***

## ﻿Discussion

Our molecular data provides compelling evidence for the existence of a new, deeply divergent, and completely sorted species within the *Perinereis* species Group I in the Red Sea. At first glance, *P.kaustiana* sp. nov. can be easily misidentified as the well-known and allegedly cosmopolitan *P.cultrifera*, due to the classic two bar shaped paragnaths in Areas VI and proximity with the Mediterranean Sea. This might be the reason the latter is usually reported for the Red Sea (Wehe and Fiege 2002; Bonyadi-Naeini et al. 2018; OBIS), but a greater sampling effort in the central and southern Red Sea regions are needed to confirm this. Morphological features, such as the paragnath arrangement, as well as the length of tentacular cirri and ratios within the parapodia also allowed the distinction of *P.kaustiana* sp. nov. from other similar species (see taxonomic key and Tables 4, 5). Upon careful morphological examination, *P.kaustiana* sp. nov. is morphologically closer to the Indo-Pacific *P.helleri*, than it is to the European *P.cultrifera*, based mainly on paragnath patterns, particularly in Areas III (Fig. 2D) and VII and VIII (Fig. 2G), and similar length of the falciger blades. Paragnath features in Areas VII and VIII lends support to the taxonomic importance of highlighting faint ridges and furrows in the ventral oral ring for certain *Perinereis* species (Conde-Vela 2018), which usually are not accounted in species descriptions due to no apparent pattern being found (i.e., Teixeira et al. 2022a). *Perinereiskaustiana* sp. nov. and *P.helleri* are also phylogenetically closely related (Fig. 1A), despite being divergent lineages, with genetic distances that are in the range used for delimitating polychaete species (i.e., Kvist 2016; Lobo et al. 2016; Nygren et al. 2018). This situation, together with the absence or subtle morphological differences previously overlooked, resembles cryptic lineages within a species complex (Teixeira et al. 2022b, 2023), and further sampling efforts between the Red Sea to the Indo-Pacific region are needed to assess this.

The new species is so far unique to the northern Red Sea and apparently easy to find in the rocky beaches of the Gulf of Aqaba. Considering the high rate of endemism in the Red Sea (DiBattista et al. 2016), this species may indeed be endemic to this Sea, although further sampling across this region and the Indo-Pacific area might prove it to be more widespread. In the remaining sampling sites further south, along the northern Saudi coast, *P.kaustiana* sp. nov. is outcompeted by the sympatric distributed *Perinereisnuntia* species group, which seems to be the dominant coastal annelid in the region (Fig. 1B). The latter is also a species complex with several different species recently revised by Villalobos-Guerrero (2019). Our specimens initially identified as belonging to the *P.nuntia* complex revealed at least two different morphotypes, which after further morphological (mainly based on paragnath patterns, Fig. 1C, D) and molecular review corresponded to the new species recently described by Elgetany et al. (2022) for the neighbouring Egyptian coast (Suez Canal), namely *P.damietta* (Fig. 1C) and *P.suezensis* (Fig. 1D). These species are sympatric with *P.kaustiana* sp. nov., but apparently not sympatric with each other in the studied region (Fig. 1B). *Perinereisdamietta* (which is morphologically more similar to *P.heterodonta* Gravier, 1899 than to *P.nuntia* according to Elgetany et al. (2022)), was found mainly in lagoon-like environments, whereas *P.suezensis* only in fully marine areas. *Perinereiskaustiana* sp. nov. shared both marine and lagoon-like habitats, with all the three sampled species found in intertidal coarse-grained sand, under rocks or cobles. As speculated by Elgetany et al. (2022), *P.damietta* seems to have a slightly wider habitat preference, since some of our specimens (from Al Muwaileh lagoon) also occurred sub-tidally, attached to small rocks at approximately 1 meter depth.

## Supplementary Material

XML Treatment for Perinereis kaustiana

## Footnotes

No available chaetae data for P. striolata.

## ﻿Additional information

### Author ORCIDs

Marcos A. L. Teixeira https://orcid.org/0000-0002-2228-2673

Chloé Julie Loïs Fourreau https://orcid.org/0000-0002-0062-2876

Juan Sempere-Valverde https://orcid.org/0000-0001-5856-9214

Susana Carvalho https://orcid.org/0000-0003-1300-1953

### Data availability

New sequence data and specimen metadata were uploaded in the project “*Perinereis* Saudi NEOM (DS-MTPNO)” within BOLD (https://v4.boldsystems.org/) and in the following link: https://dx.doi.org/10.5883/DS-MTPNO. The COI alignments (FASTA and NEXUS formats) are publicly available online at Figshare (DOI: https://www.doi.org/10.6084/m9.figshare.25097756). GenBank accession numbers: PP279002–PP279039 (mtCOI- 5P); PP264567–PP264572, PP264574, PP264575 (16S); PP264613, PP264614, PP264616 (28SD2). See Suppl. material 1 for more details. The new biological material from the Red Sea is deposited at NTNU University Museum (NTNU-VM), Trondheim, Norway. Specimens from *Perinereisoliveirae* are deposited at the Biological Research Collection (Marine Invertebrates) of the Department of Biology of the University of Aveiro (CoBI at DBUA), Portugal. All specimens are available upon request.

## Supplementary materials

Supplementary material 1 Supplementary data Data type xlsx Explanation note Voucher data, origin of the specimens and GenBank accession numbers for each of the analysed genetic markers original to this study and molecular metadata used for comparison purposes or as outgroups. https://binary.pensoft.net/file/1013368This dataset is made available under the Open Database License (http://opendatacommons.org/licenses/odbl/1.0/). The Open Database License (ODbL) is a license agreement intended to allow users to freely share, modify, and use this Dataset while maintaining this same freedom for others, provided that the original source and author(s) are credited.Marcos A. L. Teixeira, Chloé Julie Loïs Fourreau, Juan Sempere-Valverde, Susana Carvalho
